# Smoke-free or not: a pilot evaluation in selected Beijing Hospitals

**DOI:** 10.1186/1471-2458-13-964

**Published:** 2013-10-17

**Authors:** Frances A Stillman, Michelle R Kaufman, Anjie Zhen, Jingyan Yang, Jiangbo Wang, Na Zhao

**Affiliations:** 1 Department of Health, Behavior and Society, Johns Hopkins Bloomberg School of Public Health, 2213 McElderry Street, 4th floor, Baltimore, MD 21205, USA; 2 Chinese Association for Tobacco Control, Beijing, China

**Keywords:** (MeSH terms), Smoke-free, Hospitals, Evaluation, Passive nicotine monitors, Policy assessment

## Abstract

**Background:**

China enacted a policy to ban smoking in hospitals. The Chinese Association for Tobacco Control (CATC) developed a program to help hospitals implement this policy. They conducted a program and an assessment in 3 Chinese cities (Beijing, Shanghai and Guangdong). A more in-depth evaluation was implemented with a sub-sample of hospitals in Beijing (N = 7) to provide an independent assessment. This independent assessment focused on evaluating policy development and an assessment of secondhand smoke (SHS) to determine compliance with the smoke-free policy initiative.

**Methods:**

Pre- and post**-**survey data were collected at each of the selected hospitals with a total sample of 2835 physicians at pre-intervention and 2812 at post-intervention. Smoking rates pre- and post-policy implementation, change in knowledge, attitudes and practices among physicians, and compliance with policy were assessed. Measurements of airborne nicotine concentrations in selected locations in each hospital were taken: main hospital lobby; main outpatient center; emergency waiting room; and stairwell adjacent to a large inpatient ward. Hospital policies were collected, translated and rated for incorporated components necessary to implement a smoke-free policy.

**Results:**

Physicians’ smoking rates decreased and attitudes towards tobacco control improved significantly from pre-to post-intervention. Smoking was still reported in certain areas of the hospital with 96% of passive nicotine monitors as well as self-report indicating continued smoking. Nicotine levels ranged from <0.0056 to 3.94 μg/m^3^), with an overall mean of .667 μg/m^3^. Hospitals that established stronger policies seemed to have lower levels of nicotine, suggesting a relationship between policy development and compliance. This finding is interesting but just suggestive and requires further investigation to truly demonstrate if stronger policies improve compliance and produce better outcomes.

**Conclusion:**

As implementation strategies for smoke-free environments are improved and more resources are focused on hospitals, China is making progress toward achieving smoke-free hospitals. Using a model program could increase the prevalence of SHS policies across China. However, relying only on survey data may not provide an accurate assessment of this progress, and more extensive evaluation efforts are useful to understand how change can and does occur.

## Background

As the tobacco epidemic continues to evolve in China, [1,2] the burden of tobacco-related diseases continues to grow and pose tremendous challenges to the Chinese healthcare system. Tobacco use is the most important behavioral risk factor in China, which is the world’s largest grower and consumer of tobacco [1]. Smokers comprise approximately 28.1% of the Chinese population; approximately 3.5 million people (53% men and 2.4% women) smoke [3], and it is estimated that smoking-related diseases will claim more than 2 million Chinese lives annually by 2020 [4]. In addition, 67% of the Chinese population reports being exposed to secondhand smoke (SHS) in public places and 35% in workplaces [5-8]. Furthermore, studies have demonstrated high levels of SHS in hospitals, schools, governmental buildings and other workplaces across China [9-11]. By strengthening smoke-free hospital policies and providing implementation strategies, compliance could improve [12,13] while protecting the health of patients and their family members, hospital visitors, and nonsmoking healthcare workers from SHS.

In May 2009, the Ministry of Health of China, other relevant ministries and bureaus, and the Chinese Association on Tobacco Control (CATC) issued a policy document entitled, *Decisions On Introducing a National Comprehensive Smoking Ban in All Medical and Health Institutions*. This document provided details on strategies and measures to ensure that a total smoking ban will be achieved in all buildings and facilities in the health administrative sector and health institutions at all levels by 2011 [14]. CATC implemented an intervention to help hospitals develop smoke-free hospitals and educate health care professionals concerning the dangers of SHS and promote cessation [15,16].

The objective of the current paper was to conduct a pilot evaluation using environmental monitoring in a sub-sample of the hospitals in Beijing (N = 7) to provide an independent assessment as to what extent the hospitals had reached a smoke-free status. This was a pilot of a limited but more independent assessment of the level of compliance with the smoke-free policy initiative. This study combines survey data collected by CATC during a larger study with measurements of airborne nicotine concentrations in a small sample of hospitals and an assessment of policies developed by these hospitals. The nicotine concentrations in each hospital, as well as an in-depth assessment of the SHS policies each hospital developed individually were used to provide additional information concerning whether they had reached a smoke-free status.

## Methods

### Design

As part of a larger study, CATC worked with the Chinese Ministry of Health to develop guidelines for creating smoke-free hospitals. CATC then developed an intervention in the form of training materials to assist in the implementation of these guidelines in 3 cities (Beijing, Shanghai and Guangzhou). The training sessions were attended by hospital administrators and physicians in the targeted hospitals and they sought to raise awareness of reasons for a smoke-free hospital, increase knowledge of tobacco related diseases, and encourage physicians to provide smoking cessation counseling for their patients. Physicians were also encouraged to quit smoking themselves, if applicable. Following the training, hospitals were to develop their own smoke-free policies based on the guidelines provided during the training [15,16]. This paper does not focus on the effectiveness of the implementation or training activities, but rather looks at whether the self-report data was in line with the environmental nicotine measurements and policies developed.

### Hospital sample

Out of the 12 Beijing hospitals included in the CATC intervention, a sub-sample of 7 hospitals was selected for further evaluation. We decided to focus on hospitals in Beijing since a study we conducted previously to assess SHS concentrations showed that hospitals in Beijing contained the highest concentrations of nicotine when compared to hospitals in other locations in China [17]. The 7 hospitals chosen for the subsample were all Level III hospitals (having approximately 1000 beds each). These are multi-departmental hospitals that provide specialized high-level medical care and attract patients from multiple regions in China. Level III hospitals are similar to tertiary care hospitals in the U.S., and the hospitals selected are considered among the premier hospitals in China. We chose these as they serve as potential role models for other hospitals throughout the country. CATC obtained approval for the survey from the Hospital Administration Committee from each hospital. The Hospital Administrative Committee serves as their human subjects review committee and includes representatives from essential hospital administrative and medical departments. These Committees also approved the placement of the passive nicotine monitors in each hospital. The Institutional Review Board at the Johns Hopkins Bloomberg School of Public Health deemed the use of the passive nicotine monitoring as not human subjects research.

### Measures

#### Physician survey

CATC conducted anonymous cross-sectional pre-intervention and post-intervention surveys with physicians in Beijing during 2009 and 2010 [16]. Physicians were surveyed to understand their knowledge of smoking-related diseases and tobacco use, their attitudes toward creating a smoke-free hospital, and compliance with the new smoke-free policy.

#### Specific knowledge of smoking-related diseases

To assess specific harm, physicians were asked a series of questions about various diseases: lung cancer, cardiovascular disease, emphysema, stroke, sexual dysfunction, reproductive problems and osteoporosis. In addition, 12 items assessing understanding of tobacco harm were asked, including if low tar nicotine cigarettes are less harmful; if cigarette filters can reduce harm; if passive smoking causes harm; and if smoking addiction is a chronic disease. We created a scale using these items where 1 point was given for each correct answer, with possible scores ranging from 0–12.

#### Attitudes toward creating a smoke-free hospital

To assess attitudes, physicians were asked: if the hospital should ban smoking comprehensively; if physicians should set an example and not smoke; if physicians without patient interaction should be allowed to smoke indoors; and if physicians should actively offer smoking cessation to their patients. A 4-item scale was created for the attitude measures, with a point given for each response supportive of promoting a smoke-free hospital and encouraging cessation. Possible scores on the attitude scale ranged from 0–4.

#### Compliance

Physicians were asked about their own compliance with the new smoke-free policy. Physicians that self-identified as smokers were asked to indicate where they smoked before and after the policy was implemented on hospital property, including offices, bathrooms, stairwells or outdoor locations. We assessed overall reported changes in smoking behavior at each location separately.

#### Nicotine monitoring

SHS was estimated by passive sampling of vapor-phase nicotine. This methodology is described in detail elsewhere [9,17]. A total of 28 filter monitors were placed in the 7 target hospitals. The devices were installed for 7 days during the same month (August-September 2010) in 4 designated locations in each hospital: 1) main hospital lobby, 2) main outpatient center, 3) emergency waiting room, and 4) stairwell adjacent to a large inpatient ward. For each sampling device, the following data were recorded: hospital and location, date and hour when placed and removed, sampling location area, sampling location volume, and ventilation. The time-weighted average concentration of SHS in each area was estimated by passive sampling of vapor-phase nicotine using a filter badge treated with sodium bisulphate. The samplers were assembled centrally at the Exposure Assessment Facility of the Center for Urban Environmental Health, Johns Hopkins Bloomberg School of Public Health in the USA, shipped to China, and then returned at the end of the sampling period. The collected nicotine was extracted from the filter and analyzed by gas chromatography with nitrogen-selective detection. The airborne nicotine concentration (μg/m^3^) was calculated by dividing the amount of nicotine collected by the filter during the sampling period by the effective volume of air sampled (number of minutes of sampling times the effective sampling rate).

#### Policy guideline assessment

One of the main objectives of CATC’s intervention was to assist hospitals with developing a formal worksite smoking policy based on the guidelines provided. These policies, instituted in 2009, were obtained from each of the 7 target hospitals and assessed for the components included in each policy statement. As the policy guidelines were written in Chinese, the assessment was conducted by a native Chinese speaker and then reviewed by the research team. A rating schema was developed to capture the 4 over-arching components that were presented to the hospitals as policy guidelines: type of policy (100% smoke-free), accessibility and education (banning sales and advertising, designating signage requirements and hospital-wide campaigns), enforcement (such as fines and measures to promote compliance), and hospital staff intervention (training and skill development, including encouragement of cessation). Each component contained specific criteria, and hospitals were given one point for each of these criteria contained in their policy statement, for a total possible score of 13 points.

### Analysis

Survey data was analyzed using quantitative statistical analyses, while hospital policies were analyzed using qualitative content analysis. Nicotine concentrations were markedly right skewed. The limit of detection was <0.0056 μg/m^3^. The median and interquartile ranges were used to describe the data, and box plots on the logarithmic scale were used to graphically present the distribution of nicotine concentrations by hospital. To compare nicotine concentrations across hospital locations, the ratios of nicotine concentrations and its 95% CI versus the corresponding category with the lowest nicotine concentration were estimated using nicotine data that was log-transformed. The analyses were carried out with STATA 11 (StataCorp).

To explore a relationship of the policy scores with nicotine levels, four different smoothing strategies were used, including median spline, median bands, lowess plot and linear regression to do the trend estimation [18]. Both median spline and median bands models partition the data and fit separate piecewise regressions to each section, smoothing them together where they join, while *lowess* models essentially fit local polynomial regressions and join them together.

## Results

### Demographics

In 2009, a convenience sample of 2788 physicians (49.1% male, n = 1370; 50.9% female, n = 1418) at 7 hospitals were surveyed at pre-intervention (~400 per hospital; 1.7% missing data). Of those, 431 (15.5%) self-reported being smokers, and 2357 (84.5%) were non-smokers. One year after the intervention, a new sample of 2785 (49.9% male, n = 1389; 50.1% female, n = 1396) physicians were surveyed at the same hospitals (1% missing data). Among them, 295 (10.6%) identified as smokers while 2490 (89.4%) identified as non-smokers. The surveys had near equal representation of male and female physicians (chi-sq = 0.30, p = 0.58).

### Physician knowledge of smoking-related diseases

**Figure 1:**
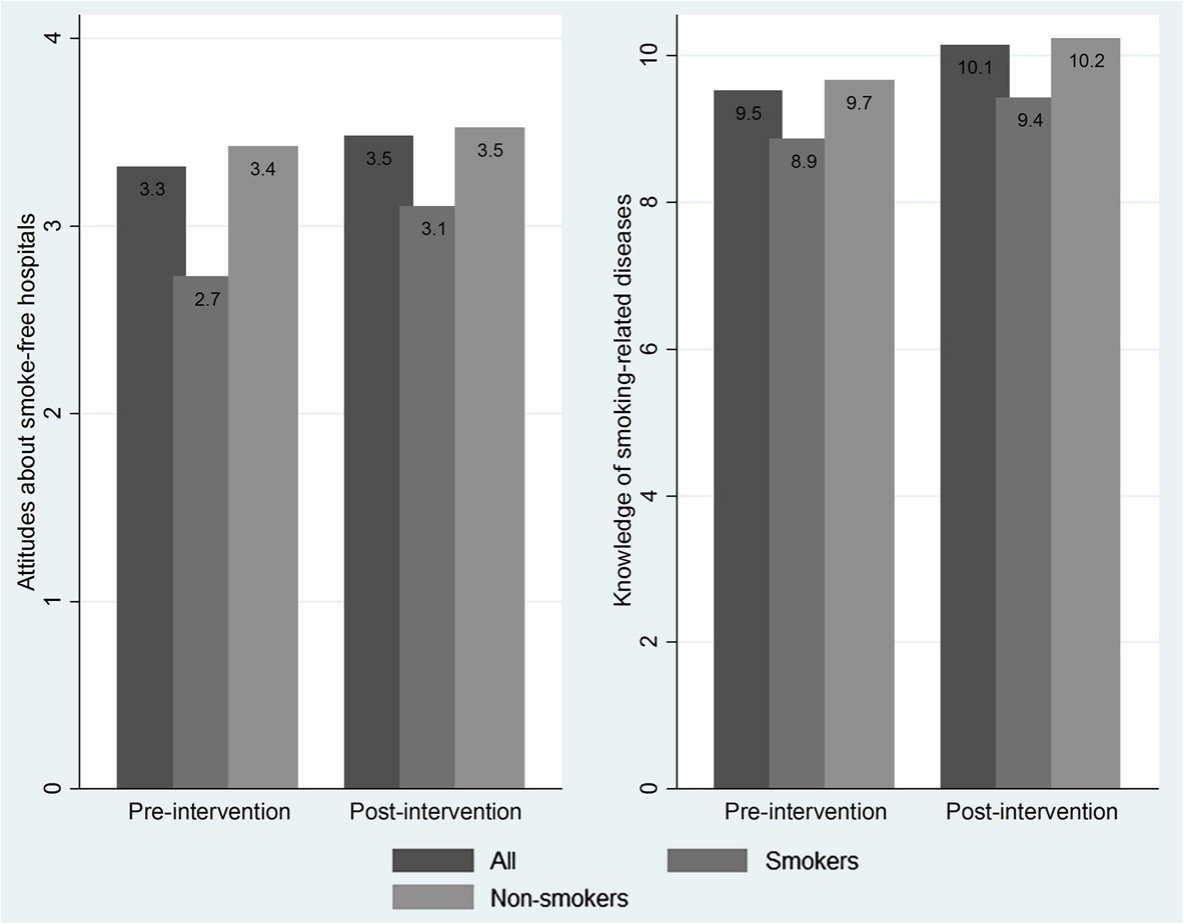
CATC intervention.

Figure 1 shows physicians’ overall specific knowledge score on tobacco related disease. This score improved slightly but significantly from pre**-**to post-intervention (9.52 to 10.15, t = −10.03, p < 0.001). Both smokers and non-smokers had a slight increase in their knowledge levels: both increasing the same amount (0.57 points). However, there was a gender difference in terms of the knowledge change. Before the intervention, female physicians scored 0.47 points higher than males (t = −4.96, p < 0.0001). After intervention, there was no statistical gender difference in knowledge scores.

### Attitudes about smoke-free hospitals

Physicians’ attitudes towards tobacco control improved significantly from pre-to post-intervention (3.31 to 3.48, t = 7.11, p < 0.001; a higher score meaning more support for smoke-free hospitals). Figure 1 shows the significant improvement in attitudes of all physicians following the intervention, as well as disaggregated for smokers and nonsmokers. Nonsmokers gained 0.1 points, a 2.9% increase over baseline (t = −4.36, p < 0.001). Smokers improved slightly more than nonsmokers in their attitudes; on average they gained 0.38 points after intervention, a 13.73% increase over baseline scores (t = −4.20, p < 0.001). No significant differences were found by gender.

### Physician compliance with smoke-free policy

**Figure 2:**
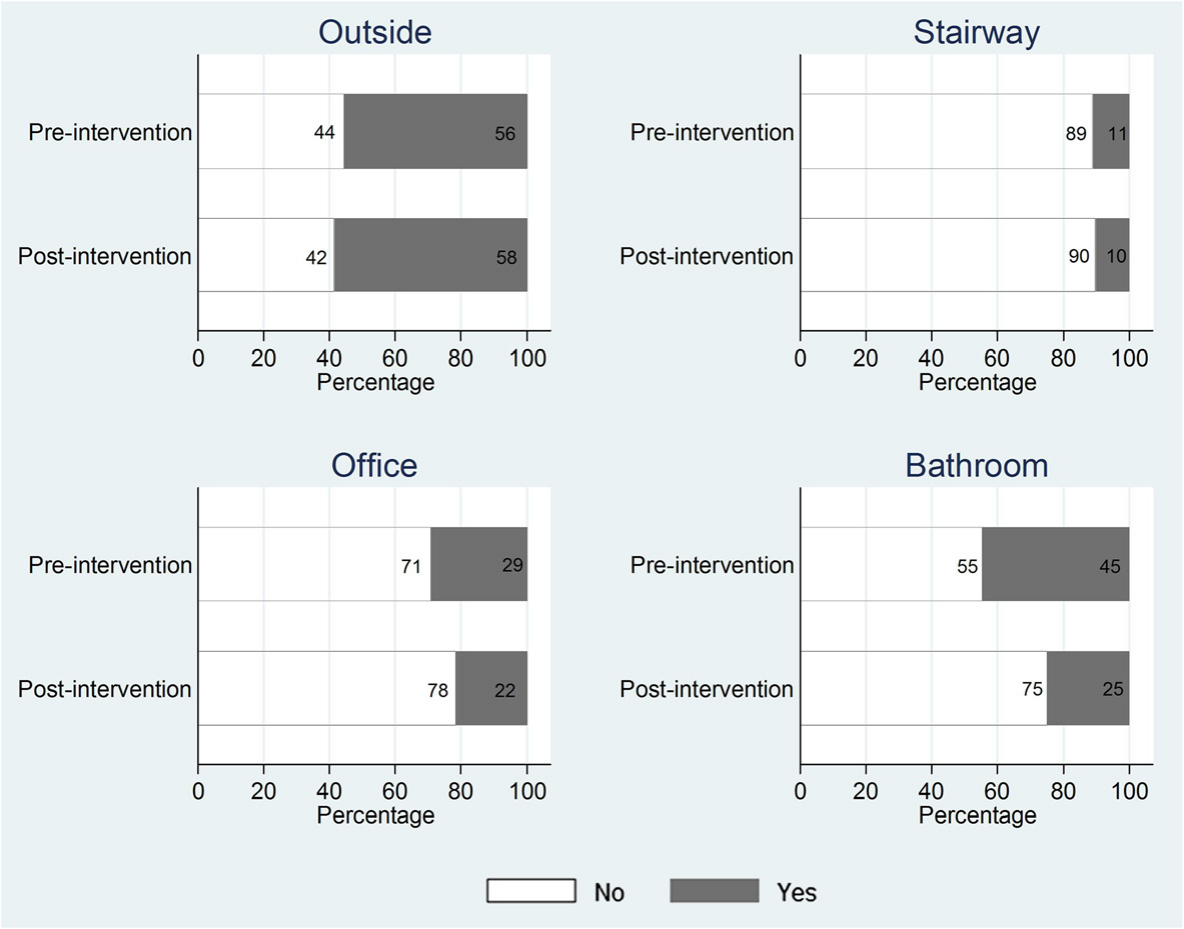
Compliance with smoke-free policy by hospital physicians.

Physicians were also asked about their compliance with the policy with regards to their own smoking behavior on hospital property. Figure 2 shows locations where physicians reported smoking before and after the smoke-free policy was instituted. After a year of policy implementation, more physicians reported they were smoking in outside locations (55.8% to 58.4%), however, the increase was not significant (chi^2^ = 0.44, p = 0.51). There was no significant difference found for physicians reporting smoking in stairwells at pre- and post**-**intervention (10.8% at baseline, 10.0% at follow-up; chi^2^ = 0.12, p = 0.73). However, significantly fewer physicians reported smoking in their offices (29.4% at baseline and 21.8% post-intervention; chi^2^ = 4.75, p = 0.03) and bathrooms (44.8% at baseline and 25.0% post-intervention; chi^2^ = 27.04, p < 0.001) following the enactment of the policy as compared to before.

### Nicotine monitoring

**Figure 3:**
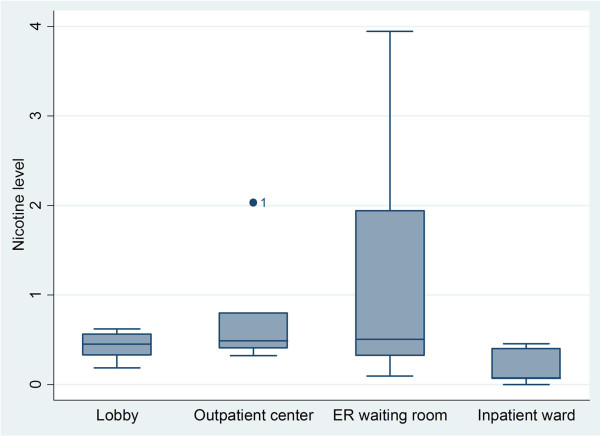
SHS passive nicotine monitor readings from 7 hospitals in Beijing.

A total of 28 filter badges (monitors) were placed in the 7 target hospitals; 25 monitors were retrieved (89% retrieval rate). Of those, 96% had detectable levels of nicotine. We observed a wide range of monitor readings, from below the level of detection, <0.0056 μg/m^3^ up to 3.94 μg/m^3^. Only 1 monitor was found that was below the level of detection. This was found in the inpatient ward at Hospital 4. The overall nicotine level across all hospitals was 0.667 μg/m^3^. The average nicotine levels for the ER waiting rooms was 1.1282 μg/m^3^, followed by the outpatient center 0.7380 μg/m^3^, the lobby 0.4359 μg/m^3^, and the inpatient ward 0.2013 μg/m^3^ (Figure 3). We detected an unusually high nicotine level (2.04 μg/m^3^, an outlier) in the outpatient center of Hospital 1 as compared to that location in other hospitals (Noted on Figure 3). The highest overall level of nicotine (3.94 μg/m3) was found in the ER of Hospital 1.

### Policy guideline assessment

Table 1 presents the scores earned by each hospital based on content analysis of their written smoke-free policy guidelines. Total scores varied from a low score of 6 points to a high score of 11 points (out of a possible 13), with the median score being 9. Only 2 of the hospitals’ policies clearly stated the smoking policy was comprehensive (100% smoke-free and included employees, patients and visitors). Other hospitals stated smoking was banned in ‘nonsmoking areas’ but lacked clear definition of what defined a smoking or nonsmoking area. Most policies were only targeted towards hospital staff. For example, 5 of 7 hospitals described consequences for noncompliance for hospital staff. Only one hospital mentioned noncompliance consequences for visitors (violators received a fine of a minimum of $2 USD), but this was not included as part of the policy score. All hospitals incorporated tobacco control performance into their department and staff evaluations, suggesting tobacco control activities were considered an important mission by all participating hospitals. Three hospitals held additional trainings for hospital staff, but only 1 hospital stated these trainings would be performed annually rather than as a one-time occurrence (not included in policy score). Five hospitals had a definitive statement banning the sale, advertising, or sponsorship of tobacco products within the hospital. Interestingly, the hospital with one of the lower policy scores (Hospital 1, which had a score of 7) had no mention of signage requirements in their policy. Hospital 3 had the lowest policy score (6) and its policy statement focused mostly on enforcement and did not include a clear statement concerning coverage of the policy or clear statements on hospital staff intervention.

**Table 1:** Analysis of hospital policy guidelines

**Hospital number**	**1**	**2**	**3**	**4**	**5**	**6**	**7**
**Policy type**							
Smoking not allowed by staff & visitors					**x**	**x**	
**Accessibility & education**							
Ban tobacco sales		**x**		**x**	**x**	**x**	**x**
Ads & sponsorship		**x**		**x**	**x**	**x**	**x**
Visible signage		**x**	**x**		**x**	**x**	**x**
Campaign and education		**x**	**x**	**x**	**x**	**x**	**x**
**Enforcement**							
Consequence of noncompliance stated	**x**		**x**	**x**		**x**	**x**
Designates supervisory duties	**x**	**x**	**x**	**x**	**x**	**x**	
Included in performance reviews	**x**	**x**	**x**	**x**	**x**	**x**	**x**
**Staff Intervention**							
Award if staff or physician quit smoking	**x**				**x**		
Physician to advise and counsel	**x**	**x**	**x**	**x**	**x**	**x**	**x**
Prohibition to smoke in uniform	**x**	**x**		**x**	**x**	**x**	**x**
Physicians prohibited from accepting		**x**		**x**	**x**		**x**
Cigarettes as gifts							
Tobacco control training	**x**			**x**		**x**	
**Score**	**7**	**9**	**6**	**10**	**11**	**11**	**9**

### Relationship between nicotine level and policy score

**Figure 4:**
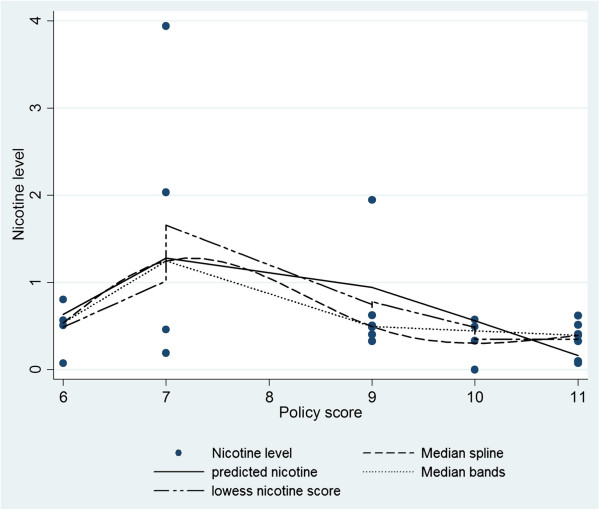
Relationship between nicotine level and policy score.

Our analysis was suggestive of a relationship between the level of nicotine measured and the policy index score (Figure 4). Overall, as the policy score increased, the measurable level of nicotine was lower. For example, the trend peaked at score 7, which was possibly due to a very high nicotine level (3.94 μg/m^3^ - an outlier) found in that hospital. Nicotine levels continuously decreased until a point, at a score around 10. After that, the nicotine level remained approximately stable over scores higher than 10. The four smoothing methods shown in the figure revealed consistency in describing the tendency mentioned above.

## Discussion

The Chinese Ministry of Health instituted a policy to require all of the country’s healthcare facilities become smoke-free. Recognizing the enormity of this goal, a mid-term objective was to achieve success in half of the countries’ hospitals by 2010 [14]. Hospitals were to implement smoke-free policies, stop selling cigarettes, and to have physicians acquire knowledge about tobacco control to support these initiatives. Guidelines were established [16], and CATC helped develop the model program, wrote guidelines for enacting the policy, and implemented the program [15]. They included pre-intervention and post–intervention surveys to assess change. However, there were expressed doubts in the media suggesting that hospitals were being designated as smoke-free when in fact they were not [17]. In order to provide additional data to better assess the progress made by CATC’s initiative, the current study provided a more in-depth evaluation implemented with a sample of the hospitals in Beijing to provide an independent assessment of the intervention’s impact. We summarized the survey data CATC collected concerning the change in attitudes, behaviors and knowledge of physicians following smoke-free policy implementation and then provided some additional measures, including environmental nicotine levels and a systematic review and assessment of written hospital smoke-free policies.

The data demonstrate progress was made to establish smoke-free facilities in selected hospitals in Beijing, providing valuable information concerning the effectiveness of the model program. Changes in knowledge about smoking occurred, positive attitudes increased, and lower levels of SHS were found. We chose Beijing for this subsample since our previous monitoring indicated Beijing had the highest passive nicotine measurements in hospitals, and we felt this would be a good example of what might be achieved by this model smoke-free hospital project [16]. For example, our previous study found levels with a range of values from 11.0 μg/m^3^ to .05 μg/m^3^. During that study, nicotine was found in 100% of the passive monitors. While we still found that 96% of the passive monitors had measurable levels of nicotine in the current study, we did see lower amounts of nicotine in our selected sub-sample of hospitals. The current study found levels of SHS in Beijing hospitals ranged from below the level of detection <0.0056 μg/m^3^ to a high of 3.94 μg/m^3^. This is a reduction of 65.1% compared to amounts found previously. The reduction is suggestive of progress since we only had our previous work in Beijing hospitals as a comparison to assess change.

Previous studies evaluating SHS exposure have also used self-reported surveys, qualitative studies, or assessment of airborne nicotine in worksites and hospitals [9,11,12,19-26]. One such study in China found compliance with smoking restriction policies was poor, with more than 40% of smokers working under a smoke-free policy reporting smoking was still occurring “sometimes” in their workplaces [21]. Evidence from focus groups conducted in 3 Chinese provinces found extensive misconceptions about SHS policies among physicians and educators [24]. In addition, hospital personnel reported that regulations had been enacted without their input, and they did not understand their responsibilities or how to contribute to implementation of the policy [24]. That study showed leadership was needed to help develop better SHS policies, improve implementation and compliance, and to provide more information to institutions to take tobacco control seriously [24].

The current CATC study was an important step to improve implementation of SHS in hospitals, as well as to understand how to develop a model program that can be disseminated across the country. By determining, in a more conclusive manner, if a sample of the hospitals had achieved a smoke-free status or made substantial progress, this study augmented CATC’s implementation by including passive nicotine monitoring and conducting an assessment of the hospital policies in addition to the survey data already collected by the organization itself. This more extensive evaluation of a sub-set of the hospitals was to help confirm results and also to determine if any link between the intervention implementation and outcomes at the hospitals could be found.

We also were able to learn more about the policies that were developed by the hospitals. We found differences between hospitals, with some producing better (more comprehensive) written policies than others. The assessment of the policies was innovative in that we categorized the content of the actual SHS policies developed by each hospital in our sample. As mentioned, we did find a range in the strength of policies, with some hospitals seemingly incorporating more of what is considered best practices into their written policy statements. However, more work still needs to be done since none of the hospitals included all of these best practices in their policy statements [16]. It was interesting to find a relationship between components incorporated in the policies and the level of nicotine found in the hospitals. It is known that more restrictive policies have the greatest impact on smoking behavior [22-27]. However, we had few observations, and one hospital had much higher nicotine levels and was classified an outlier; thus, we did not have a large enough sample to truly assess this relationship. But rather we produced an example of what could be done on a larger scale during future investigations. We also cannot ascertain which components of the policy, such as staff education vs. enforcement or signage, were most important. However, the data are suggestive that staff education may have had a smaller impact on the higher monitor readings since little was done to change smoking by patients, family and other visitors who frequent the hospitals, possibly contributing to the higher nicotine measurements.

There were also other factors that could explain some of the changes noted. In this study, a guideline was developed and training and implementation strategies were applied across all the hospitals. A pre-/post-evaluation was conducted, and the additional assessment of nicotine in the environment, as well as the assessment of the policy, provided some additional information to determine the progress of the hospitals becoming smoke-free. The results reported here are a major step forward in improving evaluation strategies used to assess the implementation of smoke-free policies in hospitals in Beijing. The results were encouraging in that physicians did have more knowledge and positive attitudes post-policy implementation, there was an indication that hospitals were no longer selling cigarettes, and smoking was occurring more in the outdoor smoking areas (albeit self-reported). However, there is evidence smoking is still occurring indoors, especially in rest rooms (self-report) and in waiting rooms (nicotine monitoring). Smoking among patients and family members is still an issue, one that was not addressed in the implementation strategies, especially as the waiting area had the highest SHS level.

While this study indicates progress has been made in moving Chinese hospitals to become smoke-free, much still needs to be done. It is evident that continued implementation is necessary to improve compliance and move the hospitals closer to becoming completely smoke-free. A plan to provide resources, including materials and funding, would help to disseminate this model as well as to assure that all hospitals, not just the large, tertiary care facilities, can develop and implement such SHS policies. In addition, more in-depth evaluation of the policies’ impact, especially through environmental monitoring such as passive nicotine monitoring, provides a more accurate assessment of the locations.

This study does have limitations. The extended evaluation was only conducted in Beijing, and only large tertiary hospitals were included. These are considered the premier hospitals in China and attract patients from all over the country, and as a consequence they potentially do not provide a generalizable view of what is happening in smaller hospitals across the country. It is also important to realize that good policy development found in a few large Beijing hospitals may not easily be disseminated to the rest of the country due to more limited resources and greater problems faced by health care facilities in other regions or cities.

We did provide an assessment of the polices as well as the pre- and post surveys that were conducted, however, we could not take into account other factors that could have influenced the changes in knowledge and attitudes among physicians. Such factors could have included media influences, other government influences, as well as the 2008 Olympics, which included a focus on health care and SHS policies. Also, we are unable to make any causal statements concerning the relationship of CATC’s initiative and any changes found since our sub-sample was small and only conducted in Beijing. Also, the survey sample was a convenience sample of physicians only, and the manner used to select the sample as well as the sample itself, could have contributed to less accurate data being collected. However, the study did try to use best practices in implementation and evaluation, which could be used in future SHS studies in China [12].

Due to increased education and knowledge among physicians, survey results may have social desirability bias. When health care professionals become more aware of what is expected in terms of smoking’s relationship to health, under reporting of smoking rates seems to be an issue. To address this, an additional evaluation component (nicotine monitoring) was useful since there was expressed concern that “many hospitals claim to be smoke-free, but in reality, they are anything but that”, and self-reported measures may not provide an accurate assessment of a smoke-free environment [17]. Using additional evaluation techniques, including nicotine measurement or systematic observation, are strategies to improve assessment of SHS compliance [9,12].

## Conclusions

As implementation strategies for smoke-free environments are improved and more resources are focused on hospitals, it is likely China will achieve the success found in other countries where hospitals have become smoke-free, physicians are more aware of the dangers of tobacco use, and smoking abstinence rates among physicians increases. This will also hopefully lead to more advocacy for SHS policies and further efforts to provide cessation advice to patients to improve the smoking situation in China. The additional evaluation provided in the current study demonstrated the model program implemented by CATC had some success and could, in fact, be used to promote SHS hospitals across China.

## Competing interests

The authors declare that they have no competing interests.

## Authors’ contributions

JW implemented the survey for CATC and assisted with the placement and return of the passive nicotine monitors. NZ conducted the analysis of the CATC survey. FS and MK developed the plan for the independent assessment and with AJ translated, reviewed and assessed the hospital policies. FS and MK wrote the 1st draft of the manuscript. FS wrote the following drafts. YJ conducted the analysis of the SHS monitor data, wrote the analysis section and developed the figures. All authors read and approved the final manuscript.

## Pre-publication history

The pre-publication history for this paper can be accessed here:

http://www.biomedcentral.com/1471-2458/13/964/prepub
